# Acute Responses and Chronic Adaptations to Cluster Versus Traditional Set Resistance Training in Males and Females

**DOI:** 10.1002/ejsc.70160

**Published:** 2026-03-14

**Authors:** Erik Hobein, Fabian Miltner, Ivan Jukic, Alexander Ferrauti, Thimo Wiewelhove

**Affiliations:** ^1^ Department of Training and Exercise Science Faculty of Sports Science Ruhr University Bochum Bochum Germany; ^2^ Department of Health Sport and Wellbeing Faculty of Social and Applied Sciences Abertay University Dundee UK; ^3^ Sport Performance Research Institute New Zealand (SPRINZ) Auckland University of Technology Auckland New Zealand; ^4^ Department of Fitness and Health IST University of Applied Sciences Düsseldorf Germany

**Keywords:** exercise, monitoring, periodisation, squat, velocity‐based training

## Abstract

This study compared acute and chronic adaptations to cluster set (CS) and traditional set (TS) structures during a 6‐week linear periodised resistance training programme in the back squat. Thirty‐six resistance‐trained females and males were randomly assigned to the CS or the TS group. Acute responses were assessed using objective (blood lactate, mean propulsive velocity, velocity loss [VL], countermovement jump [CMJ] height and modified reactive strength index) and subjective measures (rating of perceived exertion [RPE], delayed onset muscle soreness and the short recovery and stress scale). Chronic adaptations included one‐repetition maximum (1RM), relative isometric peak force, muscle endurance, CMJ height, velocity at 70% 1RM (v70) and load–velocity (L–V) profiling. CS displayed higher barbell velocities and lower acute fatigue, reflected by VL (*g* = −0.56 to −2.16), lactate (*g* = −0.51 to −1.86) and RPE (*g* = −0.91). TS did not demonstrate lower fatigue in any acute measure. Both protocols elicited comparable improvements in 1RM (CS: *g* = 0.28; TS: *g* = 0.23), muscle endurance (CS: *g* = 0.48; TS: *g* = 0.50) and v70 (CS: *g* = 1.18; TS: *g* = 1.32), with no significant improvements in CMJ height or isometric peak force. Post‐intervention L–V profiling revealed distinct adaptations, with CS demonstrating a shallower slope, indicating higher velocities at heavier loads. Sex differences were minimal; females displayed lower lactate and RPE, while longitudinal adaptations were similar. In conclusion, both protocols improved muscle strength and endurance. Collectively, CS provided superior fatigue management, better preservation of barbell velocity and unique L–V profile adaptations.

## Highlights

- Cluster sets maintained higher barbell velocities and reduced acute fatigue responses compared to traditional set structures during resistance training.
- Despite these acute differences, both cluster and traditional set trainings produced comparable improvements in muscle strength and endurance over 6 weeks.
- Cluster set training induced distinct load–velocity profile adaptations, suggesting unique neuromuscular responses and better preservation of movement quality in applied settings.

## 1 Introduction

Maintaining high movement velocity during resistance training (RT) is considered critical for enhancing power and strength adaptations (Suchomel et al. 2016; Suchomel et al. 2018). However, performing consecutive repetitions within a traditional set (TS) structure invariably leads to neuromuscular fatigue, manifested as a progressive loss of movement velocity (Tufano et al. 2016). This fatigue can compromise movement quality and may blunt the intended training stimulus. The velocity‐based approach to resistance training (VBT) has emerged as an objective method to monitor this velocity loss in real time (Guppy et al. 2024; Weakley et al. 2021). However, the practical implementation of VBT is often constrained by the cost of equipment and the technical expertise required.

This has prompted interest in alternative programming strategies that can proactively manage fatigue and maintain velocity without relying on specialised technology. One such approach is cluster set (CS) training (Jukic et al. 2022). Unlike TS structures, which are thought to drive adaptations primarily through the accumulation of metabolic stress, CS incorporates short intra‐set rests between repetitions (Suchomel et al. 2018). These rest periods, typically ranging from 10 to 30 s (Haff et al. 2008), are thought to facilitate partial phosphocreatine (PCr) resynthesis, which follows a biphasic recovery pattern. The fast component has a half‐life of approximately 20–35 s but can extend toward 50–60 s following exhaustive or high‐intensity exercise (Harris et al. 1976; McCann et al. 1995). By allowing partial PCr recovery between repetitions, CS training may help restore ATP availability, reducing the decline in force production over successive repetitions. This mechanism may enable athletes to maintain higher movement velocities and power output throughout a session, potentially promoting greater adaptations in power‐specific tasks (Suchomel et al. 2018).

Several acute studies provide evidence for these benefits, reporting that CS improves movement velocity and reduces fatigue, especially during explosive and high‐volume sessions (Tufano et al. 2016; Haff et al. 2003; Jukic et al. 2020). Despite these acute advantages, findings regarding long‐term adaptations to CS remain limited and inconsistent. Although some research suggests that CS improves muscle power and jump performance (Jukic et al. 2021), other studies report no significant difference between CS and TS for long‐term outcomes (Davies et al. 2021). The existing literature is further limited by the predominance of untrained participants, the underrepresentation of female athletes and a lack of studies directly examining the chronic effects of CS on barbell velocity at submaximal loads and jump‐related performance in trained populations.

The purpose of the present study was to compare the acute and chronic effects of CS and TS protocols during a 6‐week linear periodised RT programme centred on the free‐weight back squat. Acute responses included movement velocity, fatigue and muscle soreness, while chronic adaptations encompassed maximal strength, barbell velocity at submaximal loads, jump performance and muscle endurance. Based on previous findings and meta‐analytic evidence (Jukic et al. 2020, 2021), it was hypothesised that CS training would produce higher average movement velocities and reduced intra‐set velocity loss (VL), indicating lower acute fatigue. Although both methods were expected to elicit similar improvements in maximal strength, CS was hypothesised to yield greater gains in barbell velocity at submaximal loads and jump performance, whereas TS was anticipated to produce superior improvements in muscle endurance.

## 2 Materials and Methods

### 2.1 Participants

An a priori power analysis was conducted using G*Power (Version 3.1.9.6, University of Kiel, Germany) to estimate the required sample size to detect a medium effect size (*f* = 0.25) for changes in one‐repetition maximum (1RM) in a repeated‐measures analysis of variance (ANOVA) with two groups and two time points (pre‐ and post‐intervention). Assuming an alpha level of 0.05 and a statistical power of 0.80, the analysis indicated a required total sample size of 34 participants. Although linear mixed‐effects models (LMMs) were ultimately used for their flexibility and robustness to missing data (Sainani 2015), the initial power assumptions remained valid.

Thirty‐six strength‐trained individuals, including 15 females (age: 22.87 ± 1.96 years, height: 169.60 ± 7.08 cm, weight: 62.67 ± 4.52 kg and relative squat strength: 1.22 ± 0.13 kg per kilogramme of body mass [kg·BM^−1^]) and 21 males (age: 25.19 ± 3.04 years, height: 182.90 ± 5.40 cm, weight: 83.49 ± 9.61 kg and relative squat strength: 1.54 ± 0.24 kg·BM^−1^) participated in this study (Supporting Information S1: Table S1). Baseline relative squat strength distribution is shown in Supporting Information S1: Figure S1. Eligibility criteria were age—18–35 years, ≥ 1 year of strength training with a relative 1RM ≥ 1.00 kg·BM^−1^ (females) or ≥ 1.25 kg·BM^−1^ (males) in the back squat, absence of current injuries or illness, no use of WADA‐prohibited substances and nonpregnancy in females. Participants maintained their habitual dietary and sporting routines but avoided additional RT during the study period. The study was approved by the local ethics committee (approval number: EKS V 2024_18) and complied with the Declaration of Helsinki (WMA 2024). Written informed consent was obtained from all participants.

### 2.2 Study Design

A stratified randomised parallel‐group trial was conducted to compare the acute and chronic effects of CS training versus TS training. The study was conducted in two waves (Oct–Dec 2024 and Apr–Jun 2025) to optimise recruitment. The study lasted 8 weeks, comprising a 1‐week pretest phase, a 6‐week training intervention and a 1‐week posttest phase. During the pretest week, participants completed a familiarisation session and a baseline testing session. The training intervention consisted of twelve sessions over 6 weeks. The posttest week consisted of a single re‐evaluation of all outcome measures. Participants were matched by sex and relative 1RM strength within their respective cohort and subsequently randomised into either the CS or the TS training group using the online application *Research Randomizer* (https://randomizer.org/). A total of 19 participants (8 females and 11 males) in the CS group and 17 participants (7 females and 10 males) in the TS group completed the study and were included in the final analysis.

### 2.3 Procedures

#### 2.3.1 Familiarisation Session

A familiarisation session was conducted to familiarise participants with testing and training protocols and minimise learning effects. Body height and weight were measured in a fasted state. Participants were introduced to the standardised warm‐up protocol and to the key exercises: the free‐weight back squat, countermovement jump (CMJ) and isometric squat. Squat depth was individually assessed by having participants squat as deeply as possible with 50% of their body mass while maintaining proper form. The deepest position was recorded using a haptic barrier for consistent depth across all sessions. This individualised approach accounted for differences in mobility and muscle activation, including sex‐based differences (McKean et al. 2010; Mehls et al. 2022). The eccentric phase of the squat was performed at a self‐selected velocity, whereas the concentric phase was performed with maximum intent throughout the study. Mean propulsive velocity (MPV) was monitored using a linear position transducer (Vitruve encoder, SPEED4LIFTS S.L., Madrid, Spain) operating at a sampling frequency of 100 Hz. Knee angles for the isometric squat were set to approximately 90° using a goniometer, reflecting preferred joint angles during CMJ performance (Gheller et al. 2015). Additionally, a ten‐repetition maximum (10RM) test was conducted for the bench press, leg curl, and single‐arm cable row to determine individualised training loads for the intervention. The test began with two warm‐up sets at 50% and 60% of the estimated 1RM, followed by progressive maximal effort trials until technical or muscular failure. Rest periods of at least 3 minutes were provided between sets to ensure adequate recovery.

#### 2.3.2 Testing Sessions (Pre‐/Post‐Intervention)

The pre‐ and post‐intervention testing sessions were conducted to assess maximal strength, power and muscular endurance. Identical procedures were used pre‐ and post‐intervention.

CMJ height was assessed on force plates (ForceDecks, VALD Performance, Newstead, Australia). Participants performed three maximal CMJs with 20‐s rests, keeping their hands on their hips and descending to a self‐selected depth prior to take‐off. Jump height was calculated using the impulse–momentum method, and the average value was used for analysis, as this approach is more sensitive than peak height for detecting neuromuscular changes (Claudino et al. 2017).

Isometric squats were performed in a Smith machine (2SC Multipower, Technogym, Gambettola, Italy) with ground reaction forces measured by force plates. Participants completed three 3‐s maximal efforts at ∼90° knee flexion. The average relative peak force across three attempts, separated by 2‐min rests, were used for further analyses.

Maximal dynamic strength was determined using a 1RM test in the free‐weight back squat. The 1RM test followed a progressive loading protocol based on the participants' estimated 1RM: 5 reps at 30%, 3 reps at 50% and 70% and 1 rep at 80%, followed by incremental single attempts until 1RM was reached. Rest intervals were 2–3 min between submaximal sets and 3–5 min between maximal attempts. Proper technique was required to validate the attempt.

After ≥ 10 min of rest, participants completed three maximal repetitions at 70% of 1RM (v70) following a warm‐up set of five repetitions at 30% (v30). Average MPV across trials was analysed. Participants then performed as many repetitions as possible at 60% of 1RM. Total repetitions were recorded. At posttest, v70 and endurance tests were performed using pretest loads for direct comparison. Additional v30 and v70 trials were conducted at the new posttest 1RM loads for consistency.

#### 2.3.3 Training Sessions

Each training session followed a standardised structure: warm‐up, performance testing, squat training intervention, acute fatigue assessment and supplemental exercises.

Participants first completed the short recovery and stress scale (SRSS) (Hitzschke et al. 2015), from which the items muscular stress and overall stress were used for analysis. The standardised warm‐up was followed by CMJ testing. Participants then performed one set of five repetitions at v30 and three repetitions at v70. To account for individual variations in fatigue and adaptation, training loads were dynamically adjusted using velocity feedback. If v70 deviated by 0.07–0.14 m·s^−1^ from the baseline, estimated 1RM was adjusted by 5%; deviations ≥ 0.15 m·s^−1^ triggered a 10% adjustment. New training loads were recalculated accordingly. Participants then completed the assigned squat protocol (CS or TS). Inter‐set rest periods were 180 s, and cluster sets included additional 30 s intra‐set rests. Both groups followed a 6‐week linear periodised programme (see Table 1). Bar velocity was continuously monitored, and VL was calculated as the percentage decrease in MPV from the fastest to the last repetition in each set, averaged across sets (Sánchez‐Medina and González‐Badillo 2011).

**TABLE 1:** Detailed information about the free‐weight back squat training sessions for cluster set (CS) and traditional set (TS) groups. Rest periods are expressed as total rest per session. Inter‐set rest = 180 s; intra‐set rest for CS = 30 s.

Session	Intensity [%]	Sets × reps	TS rest [s]	CS rest [s]	CS Configuration	Undulating load in CS
1	70	3 × 8	360	450	4 + 4	No
2	70	3 × 8	360	630	2 + 2 + 2 + 2	No
3	70	3 × 8	360	540	3 + 2 + 3	67.5%, 75% and 67.5%
4	75	4 × 6	540	660	3 + 3	No
5	75	4 × 6	540	780	2 + 2 + 2	No
6	75	4 × 6	540	780	2 + 2 + 2	72.5%, 80% and 72.5%
7	80	4 × 4	540	660	2 + 2	No
8	80	4 × 4	540	900	1 + 1 + 1 + 1	No
9	80	4 × 4	540	780	1 + 1 + 2	77.5%, 85% and 77.5%
10	85	4 × 3	540	660	2 + 1	No
11	85	4 × 3	540	780	1 + 1 + 1	No
12	85	4 × 3	540	780	1 + 1 + 1	82.5%, 90% and 82.5%

**FIGURE 1:**
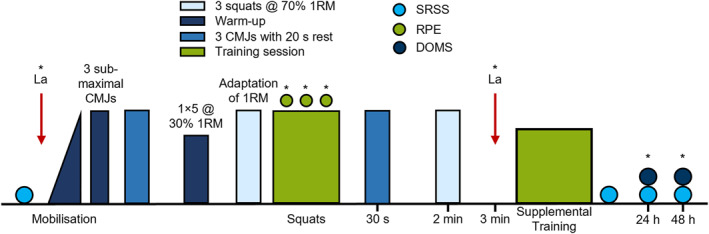
Schematic representation of the process of the training sessions. Procedures marked with * are used in Sessions 2, 5, 8 and 11. (CMJ = countermovement jump, DOMS = delayed onset muscle soreness, La = lactate, RPE = rating of perceived exertion and SRSS = short recovery and stress scale).

Thirty seconds after completing the main squat protocol, participants completed three additional CMJs, with the average jump height and the modified reactive strength index (RSI mod.), calculated as jump height divided by contraction time (Bishop et al. 2023), serving as outcome measures. In Sessions 2, 5, 8 and 11, further fatigue outcomes were also evaluated, including v70 assessments. After each squat set, participants provided their RPE, using a repetitions‐in‐reserve‐based RPE scale specific to RT (Zourdos et al. 2016). Moreover, SRSS was recorded immediately after training (post 0), as well as 24 and 48 h later. Delayed onset muscle soreness (DOMS) was also assessed at 24 and 48 h after training using a visual analogue scale (Mattacola et al. 1997). Blood lactate concentration levels were measured at rest and 3 minutes after completing the squat protocol. After the main protocol, participants performed supplemental exercises, including bench press, leg curl, single‐arm cable row and plank, to enhance adherence and standardise resistance training (Supporting Information S1: Table S2). Figure 1 schematically illustrates the sequence and structure of the full session.

### 2.4 Statistics

All statistical analyses were conducted using R (version 4.2.2; The R Foundation for Statistical Computing, Vienna, Austria). The alpha level was set at *p* < 0.05. Unless stated otherwise, descriptive statistics are presented as mean ± standard deviation. All datasets and analysis scripts are available via the Open Science Framework (https://osf.io/mej4d).

#### 2.4.1 Preliminary Analyses

Normality of dependent variables was examined via skewness and kurtosis and graphical inspection (histograms and Q–Q plots). Most variables approximated a normal distribution, except for VL, DOMS (after 24 and 48 hours) and lactate difference.

Test–retest reliability was assessed for the pretest neuromuscular measures (CMJ, v70 and relative isometric peak force) across three trials using intraclass correlation coefficients (ICC (3,k)). The ICC values were 0.99, 0.92 and 0.96, respectively, indicating excellent reliability (Koo and Li 2016). For subsequent analyses, the mean of the three trials was used to enhance measurement stability.

#### 2.4.2 Modelling Approach

LMMs were employed to account for repeated measures and interindividual variability. Unless otherwise specified, all models included fixed effects for group, session (acute effects) or time (chronic effects, including pre‐ and posttests), their interaction, and sex as a covariate with random intercepts for participants to account for individual differences. For measures with multiple postexercise timepoints (DOMS and SRSS), time was included as an additional fixed effect to account for temporal changes across measurements.

Where appropriate, three‐way interactions (group × time × sex) were tested if they significantly improved model fit (as determined via likelihood ratio tests). Model assumptions were checked via simulated residuals and graphical diagnostics. Where assumptions were violated, log transformations were applied but variables were retained only if residual diagnostics improved. Statistical significance of fixed effects was assessed using Type III ANOVA with Satterthwaite's method (Satterthwaite 1946). Where significant interactions or main effects were found, post hoc comparisons of estimated marginal means (EMMs) were conducted and adjusted for multiple comparisons via Holm's method (Holm 1979). Effect sizes (Hedges' g) (Hedges 1981) were interpreted as small (0.20), moderate (0.50) and large (0.80). (Cohen 1988).

#### 2.4.3 Acute Responses

LMMs were fitted for each primary fatigue‐related outcome (MPV, VL, changes in CMJ and RSI mod.) and secondary markers (v70 difference, lactate difference, SRSS muscular and overall stress, RPE and DOMS at Sessions 2, 5, 8 and 11).

Additionally, Spearman's rank correlations (Spearman 1904) were calculated to explore associations between all fatigue‐related outcomes across Sessions 2, 5, 8 and 11.

#### 2.4.4 Chronic Adaptations

To assess chronic training adaptations, LMMs were fitted for 1RM, relative isometric peak force, CMJ, v70 and muscular endurance (maximum repetitions). Baseline values for each respective outcome were initially included as covariates in all models to assess baseline‐adjusted change.

For CMJ and v70, this baseline‐adjusted model was retained as it improved model fit and appropriately accounted for a baseline difference in v70. Conversely, for 1RM, isometric peak force and muscular endurance, the baseline covariate was removed, as its inclusion did not improve model fit and, in some cases, worsened residual diagnostics. Sensitivity analyses confirmed that this approach did not alter the final interpretation of the results (see Supporting Information S1: Table S3). For CMJ and v70, models were fitted using an autoregressive correlation structure AR(1) to account for correlations of repeated measurements. Residual diagnostics confirmed model assumptions.

#### 2.4.5 Load–Velocity Profile Analysis

To analyse the L–V profile changes, an LMM modelled MPV as a function of load, time, group and sex, including both random intercepts and slopes for load for each participant. Minor residual deviations were tolerated due to superior model fit to alternatives. The model exhibited excellent predictive performance (conditional *R* ^2^ = 0.97 and marginal *R* ^2^ = 0.78). After evaluating fixed effects, estimated marginal trends were used to examine changes in slope over time between groups. For visualisation, regression lines were extracted from predicted values and presented alongside raw data.

## 3 Results

### 3.1 Acute Responses

**FIGURE 2:**
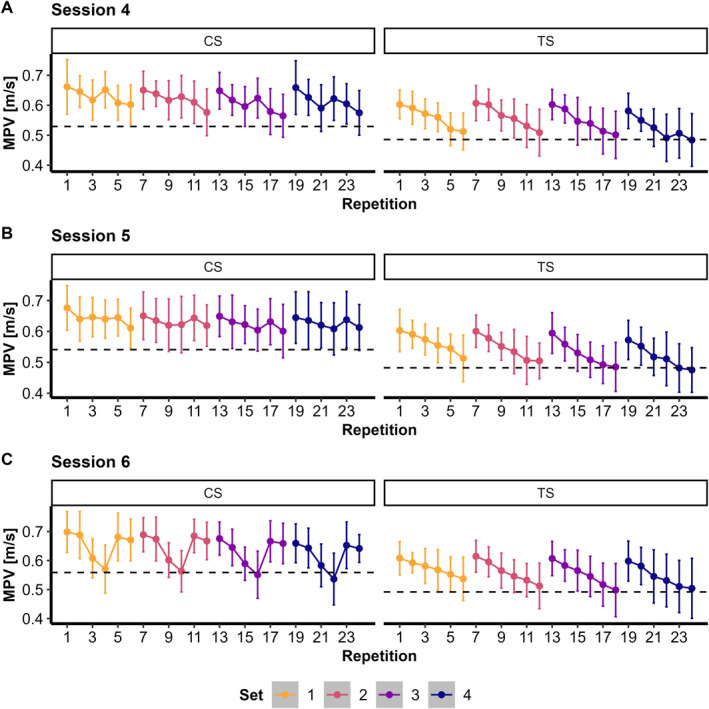
Mean propulsive velocity (MPV) presented as means ± standard deviations across training Sessions 4 (A), 5 (B) and 6 (C) for both cluster set (CS) and traditional set (TS) structures. Dashed lines indicate a 20% velocity loss (VL) threshold relative to the fastest repetition within each set.

**FIGURE 3:**
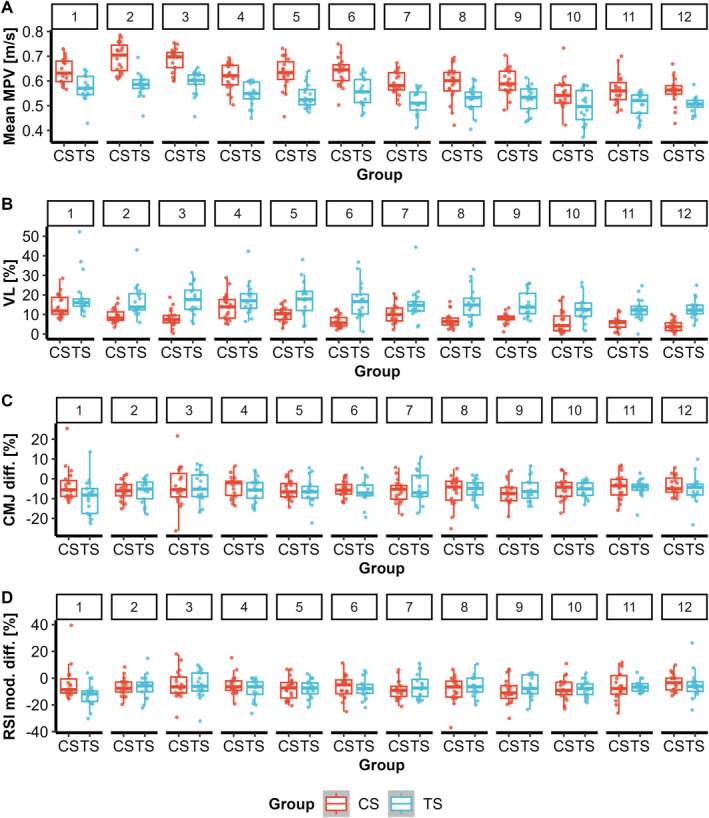
Boxplots for primary fatigue measures. Average mean propulsive velocity (MPV) (A), velocity loss (VL) (B), countermovement jump (CMJ) height difference (C) and modified reactive strength index (RSI mod.) difference (D), comparing cluster set (CS) and traditional set (TS) groups across the twelve training sessions.

The LMM for MPV revealed a significant group × session × sex interaction (*F(11, 351)* = 2.62 and *p =* 0.003), indicating that the differences between groups across sessions varied by sex. In females, the CS group consistently had higher MPV values than the TS group across all 12 training sessions (*g* = 1.33–2.49 and all *p* < 0.018) (Figures 2 and 3A, Supporting Information S1: Figures S2–S4). In males, MPV was significantly higher in CS than in TS in Sessions 1–3, 5–7 and 11 (*g* = 0.81–2.01 and all *p* < 0.046), whereas differences were not significant in Sessions 4, 8–10 and 12 (*g* = 0.29–0.76 and *p* > 0.05).

A significant group × session interaction was observed for VL (*F(11, 373)* = 3.54 and *p* < 0.001), indicating differing responses among groups across training sessions. VL was significantly lower in CS than TS in Sessions 2, 3, 5, 6 and 8–12 (*g* = −1.05 to −2.16 and all *p* < 0.004), whereas differences were not significant in Sessions 1, 4 and 7 (*g* = −0.56 to −0.67 and *p* > 0.05) (Figure 3B).

No significant interaction or main effects were found for the CMJ difference (Figure 3C) and the RSI mod. difference (Figure 3D, Supporting Information S1: Table S4) (all *p* > 0.05).

Secondary fatigue outcomes are summarised in Supporting Information S1: Table S5. Analysis of blood lactate differences revealed significant group × session interaction (*F(3, 100)* = 15.41 and *p* < 0.001) and a significant main effect for sex (*F(1, 33)* = 14.77 and *p* < 0.001). The TS group exhibited significantly higher lactate increases than the CS group at Sessions 2, 5 and 8 (all *p* < 0.001) but not at Session 11 (*p =* 0.184). Corresponding effect sizes showed moderate to large differences favouring TS (*g* = −0.51 to −1.86). Additionally, females exhibited lower overall lactate responses than males (*p =* 0.001). No significant interaction or main effects were found for v70 difference.

RPE displayed no significant interaction between group and session (*p =* 0.875) but significant main effects of group (*F(1, 33)* = 12.06 and *p =* 0.001) and sex (*F(1, 33)* = 5.54 and *p =* 0.025). RPE was lower in the CS group compared to the TS group (*g* = −0.91, *p =* 0.002). In addition, females reported lower RPE than males (*g* = −0.59, *p =* 0.025).

Analysis of DOMS revealed no significant group × session interactions (*p =* 0.959). Significant main effects of session (*F(3, 228)* = 11.02 and *p* < 0.001) and time (*F(1, 228)* = 10.07 and *p =* 0.002) were observed. DOMS was significantly higher at Sessions 2 than Sessions 8 and 11 (*p* < 0.009) and higher at Session 5 than Session 11 (*p* < 0.001). For the time factor, DOMS was significantly higher at 24 h than 48 h postexercise (*p =* 0.002).

SRSS muscular stress demonstrated a significant interaction between session and sex (*F(3, 521)* = 4.59 and *p =* 0.004), as well as a significant main effect on time (*F(3, 521)* = 35.34 and *p* < 0.001). In females, muscular stress was significantly higher at Session 5 than Sessions 8 and 11 (*p* < 0.026). In males, muscular stress was higher at Session 2 than Sessions 5 and 8 (*p* < 0.041). For the time factor, muscular stress was highest immediately postexercise (post 0) compared to pre‐exercise, 24 h, and 48 h (all *p* < 0.001), with no significant differences between the other timepoints (*p* > 0.05).

Analysis of SRSS overall stress revealed a significant group × session interaction (*F(3, 528)* = 2.73 and *p =* 0.043) and a significant main effect of time (*F(3, 527)* = 18.81 and *p* < 0.001). No significant differences between CS and TS were found at any individual session after Holm correction (*p* > 0.05). For the time factor, overall stress was highest immediately postexercise (post 0) compared to pre‐exercise, 24 h and 48 h (all *p* < 0.001), with no significant differences between the other timepoints (*p* > 0.05).

**FIGURE 4:**
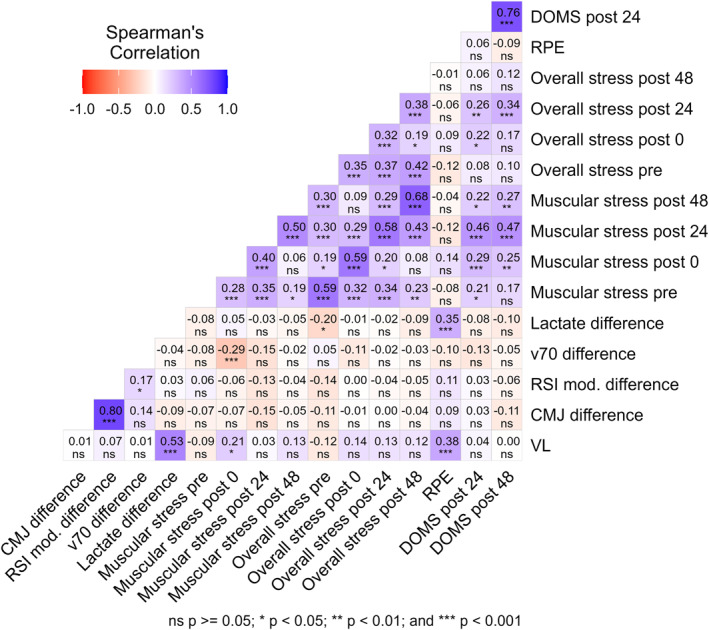
Correlation matrix of acute fatigue measures. Spearman rank correlation coefficients are shown for velocity loss (VL), countermovement jump height (CMJ) difference, modified reactive strength index (RSI mod.) difference, velocity at 70% of one‐repetition maximum (v70) difference, blood lactate concentration difference, muscular stress (pre‐, immediately post‐ and 24 and 48 h postexercise), overall stress (pre‐, immediately post‐ 24 and 48 h postexercise), ratings of perceived exertion (RPE) and delayed onset muscle soreness (DOMS) at 24 and 48 h postexercise.

Finally, Spearman correlation analyses identified that associations among fatigue measures were generally weak. Except for measures that were conceptually related (e.g., different SRSS scales or CMJ height and RSI mod. differences), most measures were either not significantly correlated or only weakly correlated (Figure 4).

### 3.2 Chronic Adaptations

Table 2 presents pre‐ and posttest descriptive data for chronic adaptations by groups. Detailed descriptive statistics for CMJ and v70 across timepoints are provided in Supporting Information S1: Table S6.

**TABLE 2:** Mean ± standard deviation of one‐repetition maximum (1RM), relative isometric peak force, muscle endurance, countermovement jump (CMJ) height and velocity at 70% of one‐repetition maximum (v70) for cluster set (CS) and traditional set (TS) groups at pre‐ and post‐intervention timepoints.

Measure	Group	Time	
		Pre‐intervention	Post‐intervention
1RM	CS	107.76 ± 31.17	116.84 ± 31.74
	TS	104.56 ± 30.70	111.91 ± 31.95
Isometric peak force	CS	25.11 ± 3.74	24.94 ± 3.74
	TS	24.28 ± 3.52	24.51 ± 3.48
Muscle endurance	CS	31.21 ± 9.88	36.53 ± 11.57
	TS	31.71 ± 9.66	36.24 ± 7.86
CMJ	CS	33.78 ± 7.45	34.04 ± 7.58
	TS	30.98 ± 7.23	31.46 ± 6.17
v70	CS	0.67 ± 0.06	0.62 ± 0.05
	TS	0.75 ± 0.08	0.69 ± 0.06

*Note:* Unit of 1RM is kg. Unit of isometric peak force is N·BM^−1^. Unit of muscle endurance is the number of repetitions. Unit of CMJ is cm. Unit of v70 is m·s^−1^.

**FIGURE 5:**
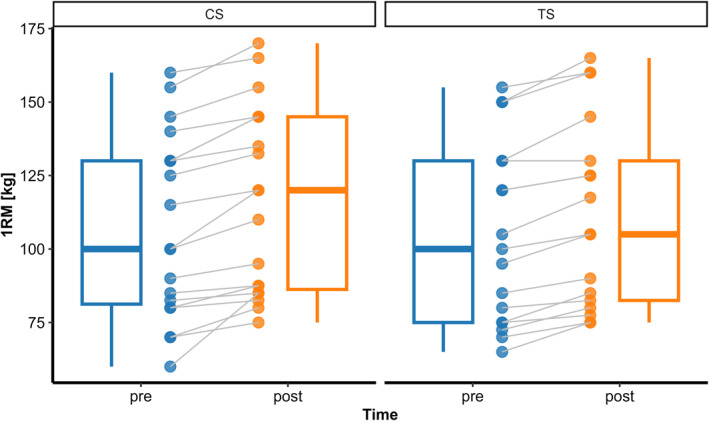
One‐repetition maximum (1RM) adaptations from pre‐to post‐intervention for cluster set (CS) and traditional set (TS) groups.

Analysis of 1RM revealed no significant time × group interaction (*p =* 0.352), but significant main effects of time (*F(1, 34)* = 80.67, *p* < 0.001) and sex (*F(1, 33)* = 95.13, *p* < 0.001) were observed. 1RM increased from pre‐ to posttest (*p* < 0.001), with small effect sizes (CS: *g* = 0.28 and TS: *g* = 0.23) (Figure 5). In addition, males demonstrated higher 1RM values than females (*p* < 0.001).

Relative isometric peak force showed no significant interactions or main effects (all *p* > 0.05) (Supporting Information S1: Figure S5A), whereas muscle endurance improved over time (*F(1, 34)* = 13.13 and *p* < 0.001) with no time × group interaction (*p =* 0.774) (Supporting Information S1: Figure S5B). In addition, a significant main effect for sex was found (*F(1, 33)* = 9.59 and *p =* 0.004). Muscle endurance improved over time (*p* < 0.001), with comparable effects for CS (*g* = 0.48) and TS (*g* = 0.50). Females were able to complete more repetitions than males (*p =* 0.004).

**FIGURE 6:**
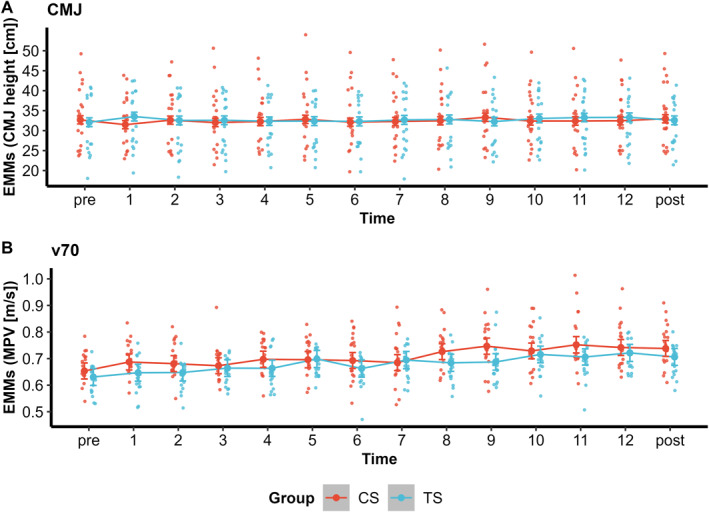
Estimated marginal means (EMMs) with 95% confidence intervals for (A) countermovement jump (CMJ) height and (B) mean propulsive velocity (MPV) at 70% of one‐repetition maximum (v70) across all timepoints for cluster set (CS) and traditional set (TS) groups.

Analysis of CMJ height showed a significant interaction between time and group (*χ* ^*2*^ *(13)* = 32.40, *p =* 0.002), but no improvements between any timepoints for both groups were observed after Holm correction (all *p* > 0.05) (Figure 6A).

For v70, a significant time × group interaction was observed (*χ* ^*2*^ *(13)* = 23.94 and *p =* 0.032).

However, post hoc analysis of the pre‐ to posttest change revealed no significant difference between the groups (*p* > 0.05), indicating that the overall magnitude of improvement was comparable. Within‐group analyses showed that v70 significantly increased from pretest to posttest in both the CS group (*p* < 0.001) and the TS group (*p* = 0.002). From pre‐ to posttest, both groups showed large effect sizes (CS: *g* = 1.18 and TS: *g* = 1.32) (Figure 6B).

### 3.3 Load–Velocity Profile

Regarding the L–V profile analysis, significant interactions were observed for load × time × group (*F(1, 432)* = 8.84 and *p =* 0.003), and load × sex (*F(1, 32)* = 36.28 and *p* < 0.001). Both CS and TS groups improved significantly from before to after at a load of 81.2 kg (all *p* < 0.001), with CS reaching significantly higher post‐training values than TS (*p =* 0.041), whereas there were no differences before training (*p =* 0.063). Males reached significantly higher velocities at a load of 81.2 kg than females (*p* < 0.001).

**FIGURE 7:**
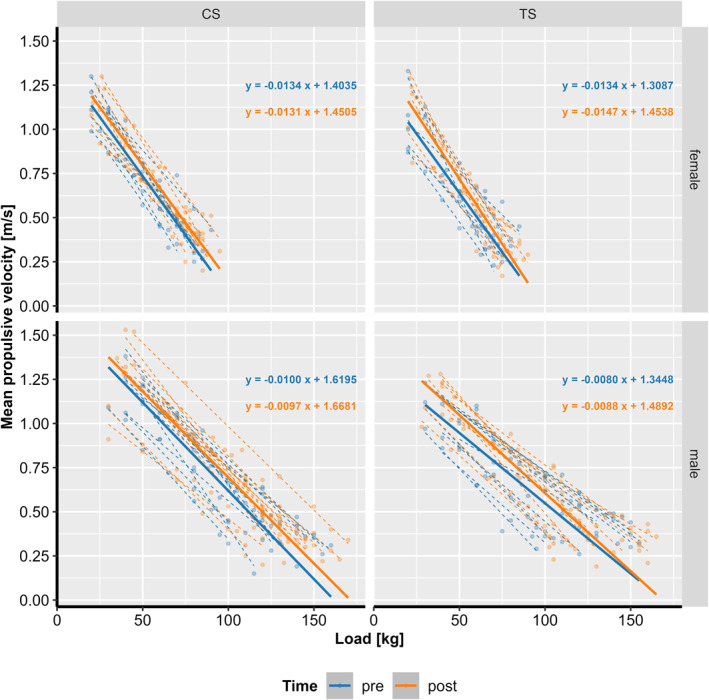
Load–velocity (L–V) profiles separated by the group (cluster set [CS] vs. the traditional set [TS]) and sex. Solid lines represent the modelled regression lines, whereas dashed lines show individual L–V profiles.

Slope analyses of the L–V relationship revealed a significant time × group interaction (*F(1, 434)* = 8.83 and *p =* 0.003), indicating that the groups adapted differently over the training period. The CS group slope remained nearly parallel from pretraining (−0.0117) to post‐training (−0.0114; *p =* 0.357), whereas the TS group exhibited a very slight steepening of the slope from pretraining (−0.0107) to post‐training (−0.0118; *p =* 0.002). These results indicate distinct load–velocity adaptations between groups (Figure 7).

## 4 Discussion

This study compared training adaptations following 6 weeks of periodised RT using either CS or TS protocols. Acute training responses were monitored session by session using objective and subjective measures to provide a comprehensive perspective on the training process. The results demonstrate that CS training consistently produced higher barbell velocities and lower acute fatigue, as indicated by MPV, VL, lactate and RPE. Rather than merely reflecting an advantage, these differences in fatigue suggest distinct stimuli for adaptation, with CS emphasising mechanical and velocity‐related stress and TS producing higher metabolic stress. Importantly, all acute fatigue measures were either similar or indicated greater fatigue in the TS group. Despite these acute differences, chronic adaptations in 1RM, relative isometric peak force, muscle endurance, CMJ height and v70 were largely similar. However, distinct post‐intervention L–V profile changes suggest different neuromuscular adaptation patterns between CS and TS trainings.

The consistent advantages of CS in maintaining higher MPV and reducing VL highlight the benefit of intra‐set rest intervals for sustaining movement quality throughout a session. These findings align with previous studies (Tufano et al. 2016; Jukic et al. 2022, 2020; Latella et al. 2019; Tufano et al. 2017) and may be explained by the short intra‐set rests that permit partial PCr resynthesis and enhanced clearance of metabolic by‐products, thereby preserving adenosine triphosphate (ATP) availability and delaying acidosis. Such mechanisms have been proposed previously (Tufano et al. 2016; Haff et al. 2008), reinforcing the utility of CS for minimising performance decline within a session. Additionally, CS training led to lower perceived and metabolic fatigue, evidenced by reduced RPE and lactate, especially during the early, high‐volume training sessions. This effect diminished during later high‐intensity and low‐volume phases, echoing previous meta‐regression findings (Jukic et al. 2020). By contrast, neuromuscular fatigue indicators (CMJ and v70 difference) and subjective fatigue indicators (SRSS and DOMS) showed no group differences. While these mixed results illustrate the complexity of fatigue monitoring, the weak correlations among different fatigue measures further emphasise the importance of adopting a multimodal monitoring strategy in practice. Furthermore, it should be noted that CS sessions took longer to complete than TS sessions due to the inclusion of additional intra‐set rest periods. This extended duration may be a practical consideration for athletes or coaches with limited time available.

Both CS and TS training elicited significant improvements in 1RM strength, muscle endurance and v70 velocity, whereas relative isometric peak force did not change significantly. Neither protocol improved CMJ height, suggesting that vertical jump performance may require longer training durations or more specific plyometric stimuli. Nonetheless, a previous study (Moreno et al. 2014) reported that CS may help maintain jump performance when incorporated into plyometric training programmes. These findings largely align with previous research indicating that CS and TS produce comparable adaptations in strength and hypertrophy (Jukic et al. 2021; Davies et al. 2021; Vargas‐Molina et al. 2025). From a physiological perspective, these similar results likely reflect the comparable total volume loads and mechanical tension achieved across both training structures. Mechanical tension is regarded as the primary driver of hypertrophy, primarily through the activation of the mammalian target of the rapamycin complex 1 (mTORC1) signalling pathway (Wackerhage et al. 2019). Although CS training enables higher barbell velocities and accordingly greater power output, this does not necessarily translate into superior long‐term adaptations when total training load is equated. Conversely, the higher metabolic stress typically observed in TS, which has also been proposed as a secondary mechanism contributing to hypertrophy (Wackerhage et al. 2019; Schoenfeld 2013), does not appear to confer a clear advantage over CS. Although some studies report superior muscle endurance following TS (Jukic et al. 2021) or enhanced CMJ and velocity outcomes with CS (Jukic et al. 2021; Oliver et al. 2013), the present results align with meta‐analytic evidence (Davies et al. 2021) suggesting both protocols are broadly effective for RT adaptations.

The L–V profile is widely used in sport science to evaluate an athlete's performance by modelling the relationship between external load and bar velocity (Guppy et al. 2024; Weakley et al. 2021). Traditionally, this relationship is examined at the individual level, often using linear regression models to estimate key parameters such as *L*_0_ (theoretical load at zero velocity) and the A line (the L–V integral based on *L*_0_ and maximal velocity capacity *v*_0_) (Iglesias‐Soler et al. 2021; Janicijevic et al. 2025). Although these approaches provide mechanistic insights, they rely on mathematically simplified assumptions, including a generalised *L*_0_, which may not capture individual differences in load adaptation over time. Another common approach is to compare velocities at specific intensities (e.g., 30%, 60% and 90% 1RM) (Pérez‐Castilla and García‐Ramos 2020), but this method does not assess how load improvements evolve over the full L–V profile. To address these limitations, this investigation employed an LMM with slope analyses to quantify changes over time and between groups. This advanced assessment of L–V profiles revealed group‐specific adaptation patterns. The CS group's slope remained largely unchanged, whereas the TS group developed a steeper slope, suggesting different neuromuscular responses to training.

The observed difference in slope adaptation may be explained by velocity specificity (Behm and Sale 1993). The TS group's steeper slope suggests that their velocity improvements did not fully transfer to the heavier end of the L–V curve. In contrast, the CS group, by consistently training at higher movement velocities (as shown by our acute data), successfully preserved their L–V relationship, resulting in significantly higher velocity at heavy loads post‐intervention. Such adaptations may enhance velocity‐specific performance outcomes, supporting CS as a strategy for improving the ability to move heavy loads quickly (García‐Ramos et al. 2020). Interestingly, these findings contrast with a previous study of Iglesias‐Soler et al. (Iglesias‐Soler et al. 2021), who observed a steeper L–V profile following rest‐redistribution sets compared to traditional sets, despite training with similarly high loads in the back squat. More recent work indicates that CS and rest‐redistribution protocols can elicit comparable adaptations in certain conditions (Janicijevic et al. 2025). For practitioners, an important insight is that the effect of CS on the L–V slope could be load‐dependent. In the present study, applying CS with heavy loads led to a shallower slope, reflecting enhanced velocity at submaximal intensities. By contrast, if CS were programmed with lighter loads, a steeper slope would be expected, as velocity improvements would dominate relative to maximal force gains. This underscores the versatility of CS as a method for tailoring L–V adaptations toward specific performance goals. Nonetheless, caution is warranted: Current evidence is mixed, and more comprehensive longitudinal studies are needed to clarify how set structures and loading strategies interact to shape the L–V profile.

Sex‐related analyses revealed broadly similar adaptations across groups, with no significant sex × time interactions for chronic performance outcomes. This aligns with previous work indicating that similar training in males and females results in similar gains in muscle hypertrophy and strength (Kojić et al. 2021; Refalo et al. 2025; Roberts et al. 2020). However, acute fatigue responses differed: Females exhibited lower RPE and blood lactate concentrations. Lower lactate values in females than males are described by existing literature (Gratas‐Delamarche et al. 1994) and are also consistent with sex‐related muscle physiology such as a higher oxidative fibre profile (Sanchez et al. 2024). In addition, it is reported that females are less fatigable (Hunter 2016), which was also demonstrated in another investigation by fewer signs of fatigue after a strength training programme with a fixed intensity and training volume compared to men (Amdi et al. 2021). Baseline sex differences were also observed, with males demonstrating greater absolute strength and CMJ values, whereas females performed more repetitions in the muscle endurance test, consistent with prior observations (Nuzzo 2023). These findings suggest that sex does not influence long‐term muscular adaptations to CS or TS training over 6 weeks. However, the observed differences in fatigue and recovery dynamics highlight the potential for sex‐specific programming considerations. For example, women may be able to tolerate higher training volumes or shorter recovery periods between sets, and auto‐regulated volume prescription could be a useful strategy to capitalise on these characteristics. Finally, future RT research should systematically include female participants, as adaptations to strength training appear to be largely comparable between the sexes, yet the literature remains male‐dominated. Eliminating this imbalance would close the sex data gap and enable more robust evidence‐based recommendations that apply to both men and women (Cowley et al. 2021).

A strength of this study lies in its dual emphasis on acute responses and chronic adaptations, offering a holistic understanding of how set structures influence both immediate fatigue and long‐term performance. The incorporation of the v70 warm‐up for daily auto‐regulation enhanced ecological validity, and the larger sample size (confirmed via G*Power analysis) increases confidence in the findings. Nonetheless, several limitations must be acknowledged. The participants differed in training experience, which may have influenced adaptation trajectories, and the 6‐week intervention may have been insufficient to detect differences in explosive outcomes such as CMJ height. Slight discrepancies between pre‐ and posttesting procedures (e.g., anthropometry and v70 placement) may also have introduced variability despite efforts to standardise conditions. Furthermore, future research should investigate VL‐matched protocols with auto‐regulated set volume and explore alternative set structures, such as rest redistribution, to further refine fatigue management strategies during RT. Finally, research including other populations, such as older adults or elite athletes, is needed to establish the generalisability of these results.

## 5 Conclusion

Both CS and TS protocols are effective for improving maximal strength and muscle endurance. However, CS offers distinct advantages in maintaining movement velocity and reducing acute fatigue, making it particularly suitable for contexts where fatigue management is critical (e.g., in‐season athletes, older adults and rehabilitation settings). Moreover, CS uniquely adapted the L–V profile by preserving higher movement velocities against heavy loads, positioning it as a practical programming strategy to achieve velocity‐specific goals, particularly in environments without access to advanced VBT monitoring equipment.

## Author Contributions

E.H., A.F., and T.W. designed the study. E.H., and F.M. conducted the study. E.H. performed the analyses and visualised the data. E.H., I.J., and T.W. interpreted the results. E.H. wrote the first draft of the manuscript. All authors edited and revised the manuscript and approved the final version of the manuscript.

## Funding

The authors have nothing to report.

## Ethics Statement

Ethical approval was obtained from the Ethics Committee of the Faculty of Sports Science of the Ruhr University Bochum (approval number: EKS V 2024_18).

## Consent

All participants were informed about the testing procedures, data policy and potential risks of the study and gave their written informed consent to voluntarily participate.

## Conflicts of Interest

The authors declare no conflicts of interest.

## Permission to Reproduce Material From Other Sources

The authors have nothing to report.

## Supporting information


Supporting Information S1

## Data Availability

The dataset and analyses code are available at the Open Science Framework (https://osf.io/mej4d).
