# Supplementary material for: Acute Responses and Chronic Adaptations to Cluster Versus Traditional Set Resistance Training in Males and Females
Source: Eur J Sport Sci. 2026 Mar 14;26(4):e70160. doi: 10.1002/ejsc.70160 (PMC13140343; doi:10.1002/ejsc.70160)
Supplement: Supplementary file 1 — Supporting Information S1 [file EJSC-26-e70160-s001.docx]

**Supplementary material**

**Table S1**

Table S1 Participant characteristics.

| **Sex** | **Age [years]** | **Body Height [cm]** | **Body Mass [kg]** | **1RM [kg]** | **rel. 1RM [kg/BM]** | **CMJ [cm]** |
| --- | --- | --- | --- | --- | --- | --- |
| female | 22.87 ± 1.96 | 169.60 ± 7.08 | 62.67 ± 4.52 | 76.00 ± 8.23 | 1.22 ± 0.13 | 26.10 ± 3.67 |
| male | 25.19 ± 3.04 | 182.90 ± 5.40 | 83.49 ± 9.61 | 127.86 ± 20.22 | 1.54 ± 0.24 | 37.00 ± 5.81 |

**Table S2**

Table S2 Supplemental training.

| **Exercise** | **Sessions 1 to 3** | **Sessions 4 to 6** | **Sessions 7 to 9** | **Sessions 10 to 12** |
| --- | --- | --- | --- | --- |
| Bench press | 3×8 @ 90% 10RM, 120 seconds rest | 3×8 @ 95% 10RM, 120 seconds rest | 3×6 @ 10RM, 120 seconds rest | 3×6 @ 105% 10RM, 120 seconds rest |
| Leg curl | 3×8 @ 90% 10RM, 120 seconds rest | 3×8 @ 95% 10RM, 120 seconds rest | 3×6 @ 10RM, 120 seconds rest | 3×6 @ 105% 10RM, 120 seconds rest |
| Single arm cable row | 3×8 @ 90% 10RM, 120 seconds rest | 3×8 @ 95% 10RM, 120 seconds rest | 3×6 @ 10RM, 120 seconds rest | 3×6 @ 105% 10RM, 120 seconds rest |
| Plank | 2×30 seconds @ BM, 60 seconds rest | 3×30 seconds @ BM, 60 seconds rest | 2×45 seconds @ BM, 60 seconds rest | 3×45 seconds @ BM, 60 seconds rest |
| Side plank | 2×30 seconds @ BM, 60 seconds rest | 3×30 seconds @ BM, 60 seconds rest | 2×45 seconds @ BM, 60 seconds rest | 3×45 seconds @ BM, 60 seconds rest |

**Table S3 (sensitivity analysis)**

To ensure robustness of the main findings, sensitivity analyses were conducted for all chronic performance outcomes (1RM, isometric peak force, muscle endurance, CMJ, v70). Specifically, whether including baseline performance as a covariate or applying log-transformation altered the results. These analyses aimed to verify that potential baseline imbalances or deviations from model assumptions did not bias the main conclusions.

For each outcome, two models were fitted:

1. Main model (as reported in the Results section): fixed effects for *Time, Group, Sex, and their interaction(s)*, random intercept for ID (and AR(1) correlation for CMJ and v70).
2. Baseline-adjusted model: identical specification but including the baseline value of the outcome as a covariate.

Table S3 Sensitivity analyses comparing the main model and the baseline-adjusted model. Outcome measures include one-repetition maximum (1RM), isometric peak force, maximal repetitions (Max reps), countermovement jump (CMJ) height, and mean velocity at 70% of 1RM (v70). For CMJ and v70, models accounted for a first-order autoregressive correlation structure (AR(1)).

| **Outcome** | **Model** | **Time × Group (*p*)** | **Group (*p*)** | **Time (*p*)** | **Sex (*p*)** | **Baseline (*p*)** |
| --- | --- | --- | --- | --- | --- | --- |
| 1RM | Main | 0.352 | 0.403 | <0.001 | <0.001 | – |
|  | + Baseline | 0.343 | 0.270 | <0.001 | 0.071 | <0.001 |
| Isometric peak force | Main | 0.462 | 0.572 | 0.901 | 0.074 | – |
|  | + Baseline | 0.454 | 0.511 | 0.900 | 0.182 | <0.001 |
| Max reps | Main | 0.774 | 0.947 | <0.001 | 0.004 | – |
|  | + Baseline | 0.766 | 0.830 | <0.001 | 0.353 | <0.001 |
| CMJ | Main (AR1) | 0.002 | 0.020 | 0.046 | <0.001 | – |
|  | + Baseline (AR1) | 0.002 | 0.401 | 0.062 | 0.774 | <0.001 |
| v70 | Main (AR1) | 0.037 | 0.042 | <0.001 | 0.337 | – |
|  | + Baseline (AR1) | 0.033 | 0.280 | <0.001 | 0.456 | <0.001 |

Model assumptions worsened for 1RM, isometric peak force, and muscle endurance when baseline was included, as indicated by singular fit warnings and deteriorated residual diagnostics, despite lower AIC values. For CMJ and v70, baseline adjustment improved model fit (lower AIC, stable residuals), yet results remained substantively unchanged. Sensitivity analyses therefore confirmed the robustness of all main findings.

**Table S4**

Table S4 Acute effects on mean propulsive velocity (MPV), velocity loss (VL), counter movement jump (CMJ) difference, and modified reactive strength index (RSI mod.) difference. Values for cluster set (CS) and traditional set (TS) training groups are presented as mean ± standard deviation, *p* values for Holm corrected post-hoc tests and Hedges’ *g* corrected effect sizes.

| **Session** | **MPV** | | | |  | **VL** | | | |  | **CMJ difference** | | | |  | **RSI mod. difference** | | | |
| --- | --- | --- | --- | --- | --- | --- | --- | --- | --- | --- | --- | --- | --- | --- | --- | --- | --- | --- | --- |
|  | **CS** | **TS** | ***p*** | **Hedges’ g** |  | **CS** | **TS** | ***p*** | **Hedges’ g** |  | **CS** | **TS** | ***p*** | **Hedges’ g** |  | **CS** | **TS** | ***p*** | **Hedges’ g** |
| 1 | 0.64 ± 0.05 | 0.57 ± 0.05 | <0.001 | 1.34 |  | 14.27 ± 5.90 | 19.52 ± 11.13 | 0.133 | –0.64 |  | –3.13 ± 8.60 | –9.18 ± 8.99 | 0.006 | 0.67 |  | –4.07 ± 12.55 | –12.40 ± 8.94 | 0.004 | 0.75 |
| 2 | 0.70 ± 0.06 | 0.58 ± 0.05 | <0.001 | 2.02 |  | 9.41 ± 3.70 | 17.32 ± 8.44 | 0.002 | –1.37 |  | –5.82 ± 4.98 | –5.97 ± 5.94 | 0.944 | 0.03 |  | –6.55 ± 6.70 | –6.48 ± 8.97 | 0.981 | –0.01 |
| 3 | 0.69 ± 0.05 | 0.59 ± 0.05 | <0.001 | 1.86 |  | 8.22 ± 4.33 | 17.60 ± 7.71 | <0.001 | –1.37 |  | –3.81 ± 10.14 | –4.19 ± 7.37 | 0.858 | 0.04 |  | –4.88 ± 10.72 | –5.20 ± 10.35 | 0.913 | 0.03 |
| 4 | 0.62 ± 0.05 | 0.55 ± 0.05 | <0.001 | 1.34 |  | 14.04 ± 6.63 | 18.18 ± 8.46 | 0.146 | –0.56 |  | –3.88 ± 5.51 | –5.57 ± 6.11 | 0.483 | 0.28 |  | –4.90 ± 7.59 | –8.77 ± 7.85 | 0.206 | 0.49 |
| 5 | 0.63 ± 0.07 | 0.54 ± 0.05 | <0.001 | 1.56 |  | 9.89 ± 3.58 | 18.26 ± 8.75 | 0.004 | –1.05 |  | –5.61 ± 4.96 | –6.34 ± 6.84 | 0.784 | 0.12 |  | –7.75 ± 8.12 | –7.76 ± 6.67 | 0.994 | <0.01 |
| 6 | 0.64 ± 0.06 | 0.56 ± 0.06 | <0.001 | 1.35 |  | 6.42 ± 3.13 | 16.95 ± 9.19 | <0.001 | –1.45 |  | –5.47 ± 4.27 | –6.48 ± 6.32 | 0.690 | 0.18 |  | –6.42 ± 8.66 | –7.61 ± 7.49 | 0.682 | 0.14 |
| 7 | 0.59 ± 0.05 | 0.52 ± 0.05 | <0.001 | 1.52 |  | 10.50 ± 5.00 | 15.48 ± 8.84 | 0.053 | –0.67 |  | –6.24 ± 5.69 | –4.03 ± 8.06 | 0.286 | –0.31 |  | –8.82 ± 7.01 | –6.06 ± 9.24 | 0.353 | –0.33 |
| 8 | 0.59 ± 0.07 | 0.53 ± 0.05 | <0.001 | 0.97 |  | 7.00 ± 4.02 | 15.86 ± 8.34 | <0.001 | –1.37 |  | –6.33 ± 7.70 | –5.30 ± 5.07 | 0.572 | –0.15 |  | –8.72 ± 10.00 | –6.23 ± 7.72 | 0.383 | –0.27 |
| 9 | 0.60 ± 0.05 | 0.53 ± 0.05 | <0.001 | 1.23 |  | 7.96 ± 2.78 | 15.21 ± 6.57 | 0.001 | –1.39 |  | –7.30 ± 5.47 | –5.54 ± 6.08 | 0.359 | –0.3 |  | –9.71 ± 9.46 | –6.40 ± 8.34 | 0.237 | –0.36 |
| 10 | 0.55 ± 0.06 | 0.50 ± 0.07 | 0.001 | 0.79 |  | 6.11 ± 5.64 | 12.73 ± 6.10 | <0.001 | –1.19 |  | –4.84 ± 5.65 | –5.58 ± 4.03 | 0.730 | 0.15 |  | –7.84 ± 8.26 | –7.72 ± 6.07 | 0.969 | –0.02 |
| 11 | 0.56 ± 0.06 | 0.50 ± 0.05 | 0.001 | 1.09 |  | 5.54 ± 3.15 | 12.61 ± 5.69 | <0.001 | –1.07 |  | –3.95 ± 6.33 | –4.60 ± 4.59 | 0.759 | 0.12 |  | –5.99 ± 10.47 | –6.11 ± 4.49 | 0.952 | 0.02 |
| 12 | 0.56 ± 0.06 | 0.50 ± 0.03 | 0.002 | 1.19 |  | 4.05 ± 2.72 | 12.99 ± 5.10 | <0.001 | –2.16 |  | –3.52 ± 4.78 | –5.01 ± 7.17 | 0.424 | 0.24 |  | –3.25 ± 6.40 | –4.86 ± 10.84 | 0.522 | 0.18 |
| Note. Unit of MPV is m·s^-1^. Unit of VL, CMJ and RSI mod. difference is %. | | | | | | | | | | | | | | | | | | | |

**Table S5**

Table S5 Descriptive statistics for secondary fatigue measure across training sessions 2, 5, 8, and 11 for cluster set (CS) and traditional set (TS) groups, presented as mean ± standard deviation. Measures include mean velocity at 70% of one-repetition maximum (v70) difference, blood lactate difference, muscular stress, overall stress, ratings of perceived exertion (RPE), and delayed onset muscle soreness (DOMS).

| **Measure** | **Group** | **Session** | | | |
| --- | --- | --- | --- | --- | --- |
|  |  | **2** | **5** | **8** | **11** |
| v70 difference | CS | –1.84 ± 7.06 | –2.23 ± 8.29 | –4.09 ± 8.59 | –3.48 ± 7.87 |
|  | TS | 1.46 ± 11.37 | –2.58 ± 4.99 | 1.70 ± 3.70 | –3.63 ± 5.49 |
| Lactate difference | CS | 0.56 ± 0.73 | 0.63 ± 0.67 | 0.31 ± 0.50 | 0.41 ± 0.45 |
|  | TS | 2.63 ± 1.98 | 1.87 ± 1.36 | 1.15 ± 1.01 | 0.71 ± 0.61 |
| Muscular stress pre | CS | 3.10 ± 1.24 | 2.21 ± 1.08 | 2.42 ± 1.12 | 2.17 ± 1.38 |
|  | TS | 2.18 ± 1.33 | 1.65 ± 1.22 | 2.29 ± 1.26 | 1.94 ± 1.34 |
| Muscular stress post 0 | CS | 3.63 ± 1.30 | 3.47 ± 1.07 | 3.05 ± 1.35 | 3.44 ± 1.50 |
|  | TS | 3.71 ± 1.40 | 3.29 ± 1.16 | 3.53 ± 1.23 | 3.41 ± 1.06 |
| Muscular stress post 24 | CS | 2.95 ± 1.13 | 2.90 ± 1.24 | 2.37 ± 1.38 | 2.28 ± 1.36 |
|  | TS | 2.53 ± 1.62 | 2.88 ± 1.50 | 1.94 ± 1.09 | 2.18 ± 1.24 |
| Muscular stress post 48 | CS | 2.37 ± 1.42 | 2.05 ± 1.08 | 2.05 ± 1.18 | 1.78 ± 1.48 |
|  | TS | 2.12 ± 1.50 | 2.65 ± 1.50 | 1.94 ± 0.90 | 2.59 ± 1.62 |
| Overall stress pre | CS | 2.53 ± 1.07 | 2.26 ± 1.24 | 2.42 ± 1.22 | 2.50 ± 1.25 |
|  | TS | 2.41 ± 1.28 | 1.82 ± 1.01 | 2.53 ± 1.33 | 2.35 ± 1.50 |
| Overall stress post 0 | CS | 3.53 ± 1.31 | 3.21 ± 1.32 | 2.84 ± 1.21 | 3.28 ± 1.18 |
|  | TS | 2.77 ± 1.52 | 3.12 ± 0.86 | 3.47 ± 1.38 | 3.65 ± 1.17 |
| Overall stress post 24 | CS | 2.74 ± 1.10 | 3.10 ± 1.33 | 2.10 ± 1.29 | 2.06 ± 1.35 |
|  | TS | 2.35 ± 1.58 | 2.65 ± 1.66 | 2.41 ± 1.42 | 2.41 ± 1.28 |
| Overall stress post 48 | CS | 2.37 ± 1.21 | 2.21 ± 1.27 | 2.00 ± 1.16 | 2.39 ± 1.24 |
|  | TS | 2.29 ± 1.69 | 2.71 ± 1.57 | 2.29 ± 1.36 | 2.88 ± 1.50 |
| RPE | CS | 6.10 ± 1.20 | 6.38 ± 1.34 | 6.05 ± 1.84 | 6.13 ± 1.32 |
|  | TS | 7.28 ± 1.37 | 7.54 ± 0.94 | 7.35 ± 1.05 | 7.11 ± 1.03 |
| DOMS post 24 | CS | 35.18 ± 22.92 | 31.18 ± 16.93 | 22.00 ± 17.55 | 17.06 ± 15.27 |
|  | TS | 26.29 ± 23.61 | 23.65 ± 19.64 | 20.65 ± 19.94 | 16.12 ± 21.24 |
| DOMS post 48 | CS | 29.59 ± 23.06 | 23.82 ± 17.97 | 18.94 ± 15.53 | 15.13 ± 15.51 |
|  | TS | 19.59 ± 18.07 | 19.41 ± 21.38 | 17.77 ± 20.51 | 15.65 ± 21.44 |
| Note. Unit of v70 and Lactate difference is %. Unit of all other variables are AU. | | | | | |

**Table S6**

Table S6 Descriptive statistics for countermovement jump (CMJ) height and mean velocity at 70% of one-repetition maximum (v70) for cluster set (CS) and traditional set (TS) groups, presented as mean ± standard deviation.

| **Time** |  | **CMJ** | |  | **v70** | |
| --- | --- | --- | --- | --- | --- | --- |
|  |  | **CS** | **TS** |  | **CS** | **TS** |
| pre |  | 33.78 ± 7.45 | 30.98 ± 7.23 |  | 0.67 ± 0.06 | 0.62 ± 0.05 |
| 1 |  | 32.57 ± 6.31 | 32.40 ± 7.17 |  | 0.70 ± 0.06 | 0.63 ± 0.07 |
| 2 |  | 33.69 ± 7.09 | 31.66 ± 7.51 |  | 0.70 ± 0.06 | 0.63 ± 0.06 |
| 3 |  | 33.08 ± 7.51 | 31.50 ± 7.22 |  | 0.69 ± 0.07 | 0.65 ± 0.05 |
| 4 |  | 33.36 ± 7.14 | 31.36 ± 6.29 |  | 0.71 ± 0.07 | 0.65 ± 0.07 |
| 5 |  | 33.93 ± 7.93 | 31.22 ± 6.63 |  | 0.71 ± 0.07 | 0.68 ± 0.05 |
| 6 |  | 33.20 ± 7.57 | 31.14 ± 6.56 |  | 0.71 ± 0.09 | 0.65 ± 0.06 |
| 7 |  | 33.35 ± 7.05 | 31.53 ± 7.54 |  | 0.70 ± 0.10 | 0.68 ± 0.06 |
| 8 |  | 33.49 ± 7.57 | 31.69 ± 7.20 |  | 0.74 ± 0.07 | 0.67 ± 0.06 |
| 9 |  | 34.19 ± 8.06 | 31.52 ± 6.71 |  | 0.75 ± 0.10 | 0.68 ± 0.08 |
| 10 |  | 33.43 ± 7.06 | 32.00 ± 6.69 |  | 0.74 ± 0.08 | 0.70 ± 0.07 |
| 11 |  | 32.85 ± 7.96 | 32.37 ± 7.07 |  | 0.77 ± 0.11 | 0.69 ± 0.07 |
| 12 |  | 33.54 ± 6.89 | 31.66 ± 6.57 |  | 0.75 ± 0.09 | 0.71 ± 0.06 |
| post |  | 34.04 ± 7.58 | 31.46 ± 6.17 |  | 0.75 ± 0.08 | 0.69 ± 0.06 |
| Note. Unit of CMJ is cm. Unit of v70 is m·s⁻¹. | | | | | | |

**Figure S1**


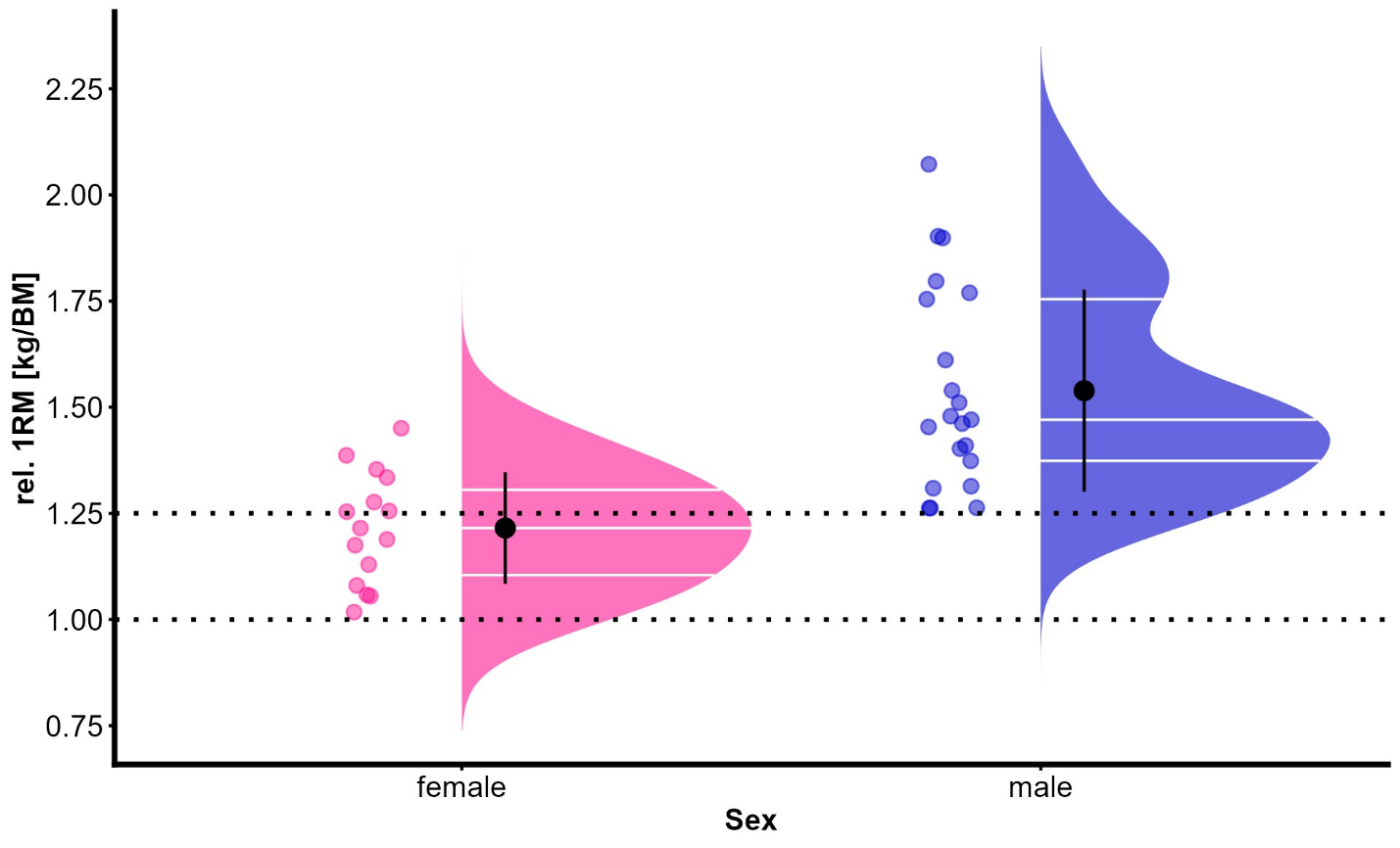


Figure S1 Distribution of participants’ relative one-repetition maximum (1RM) strength levels in the free-weight back squat during the pre-test.

**Figure S2**


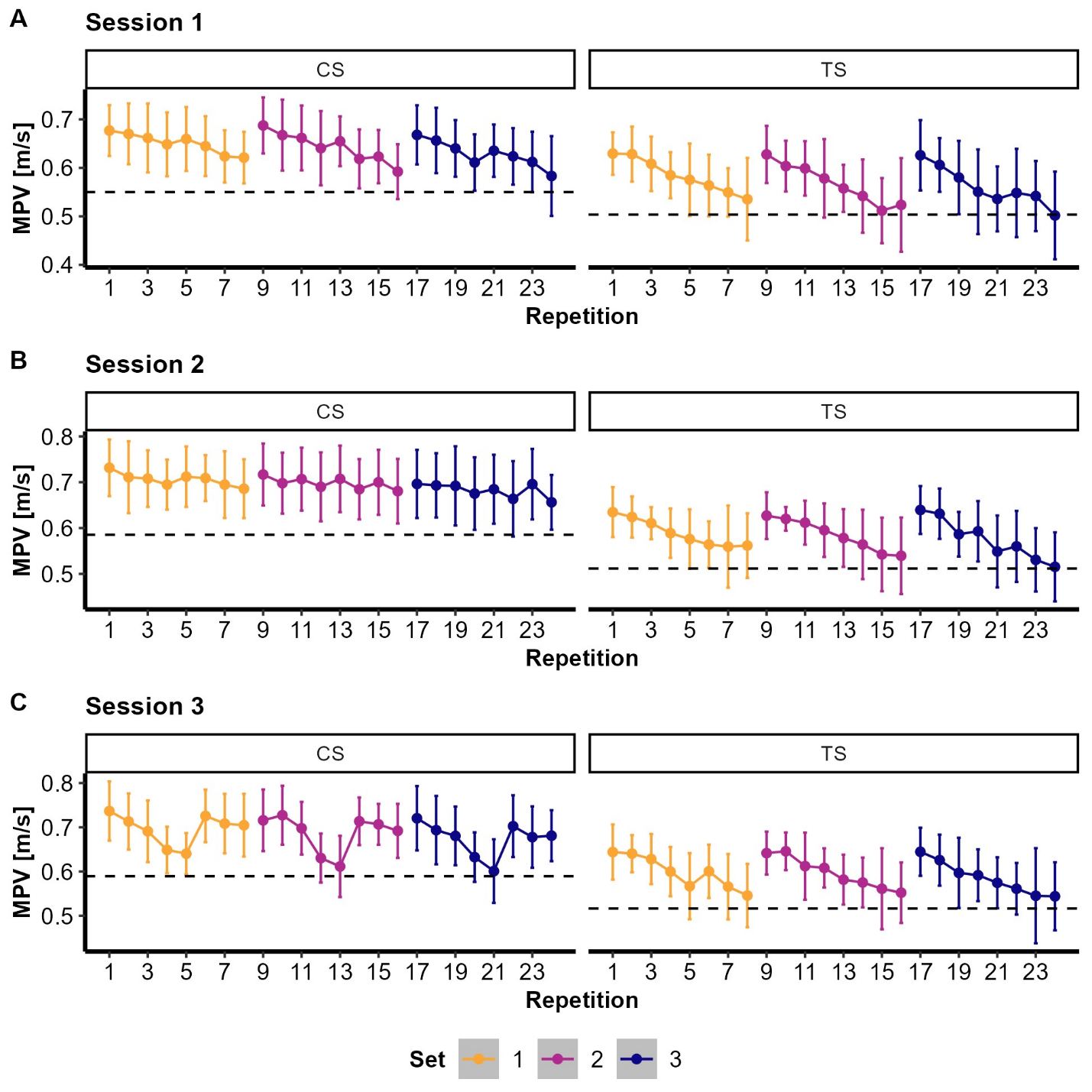


Figure S2 Mean propulsive velocity (MPV) presented as means ± standard deviations across training sessions 1 (A), 2 (B), and 3 (C) for both cluster set (CS) and traditional set (TS) structures. Dashed lines indicate a 20% velocity loss (VL) threshold relative to the fastest repetition within each set.

**Figure S3**


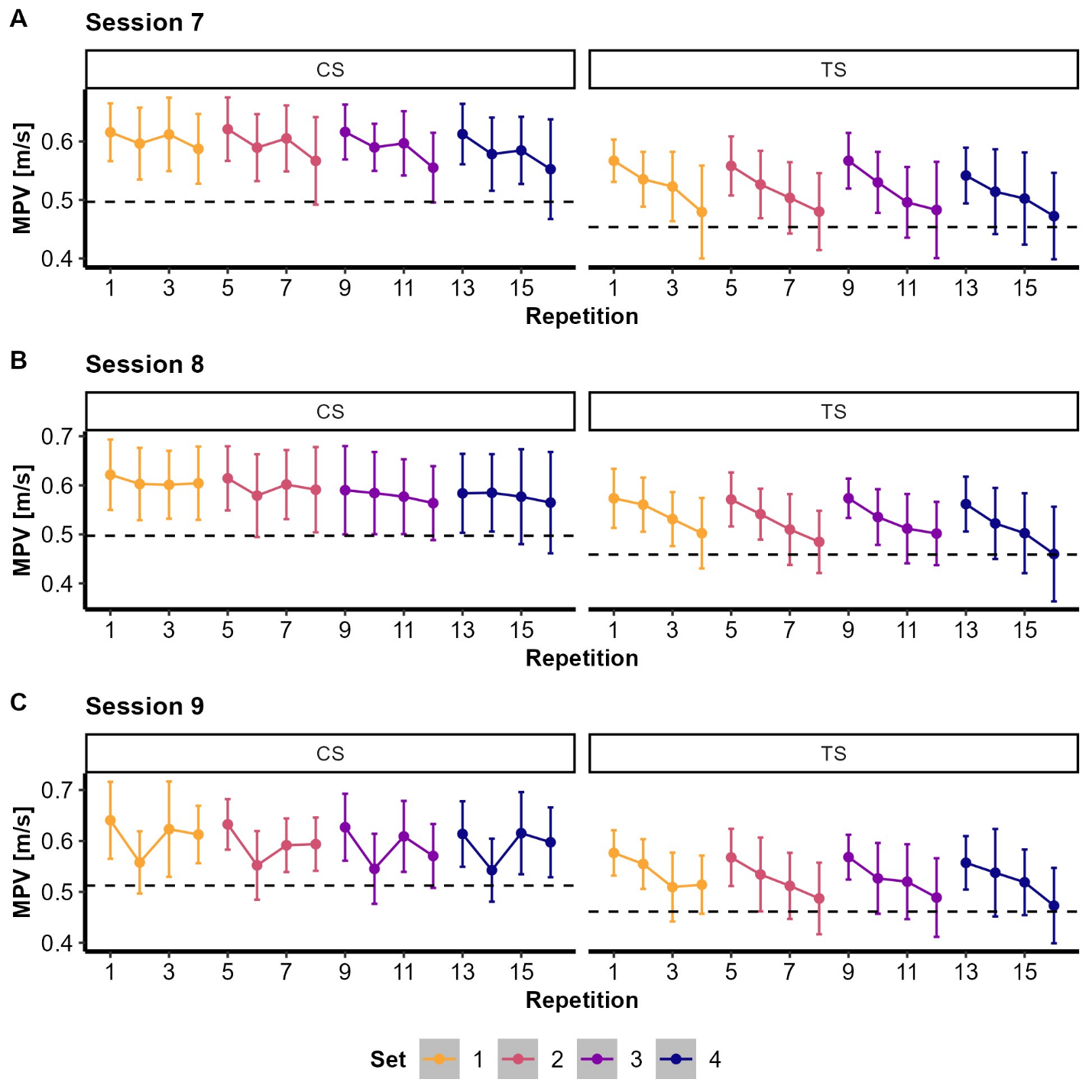


Figure S3 Mean propulsive velocity (MPV) presented as means ± standard deviations across training sessions 7 (A), 8 (B), and 9 (C) for both cluster set (CS) and traditional set (TS) structures. Dashed lines indicate a 20% velocity loss (VL) threshold relative to the fastest repetition within each set.

**Figure S4**


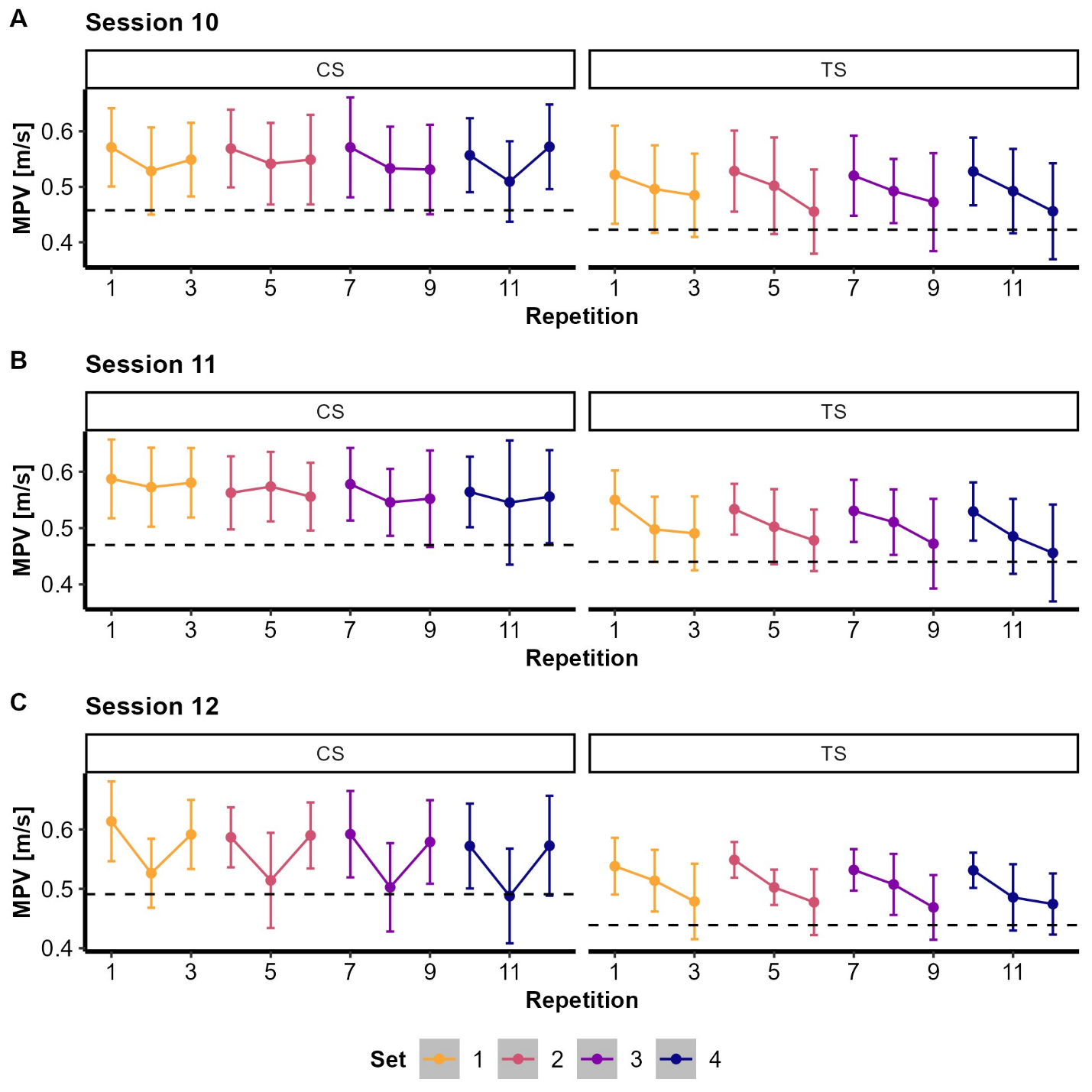


Figure S4 Mean propulsive velocity (MPV) presented as means ± standard deviations across training sessions 10 (A), 11 (B), and 12 (C) for both cluster set (CS) and traditional set (TS) structures. Dashed lines indicate a 20% velocity loss (VL) threshold relative to the fastest repetition within each set.

**Figure S5**


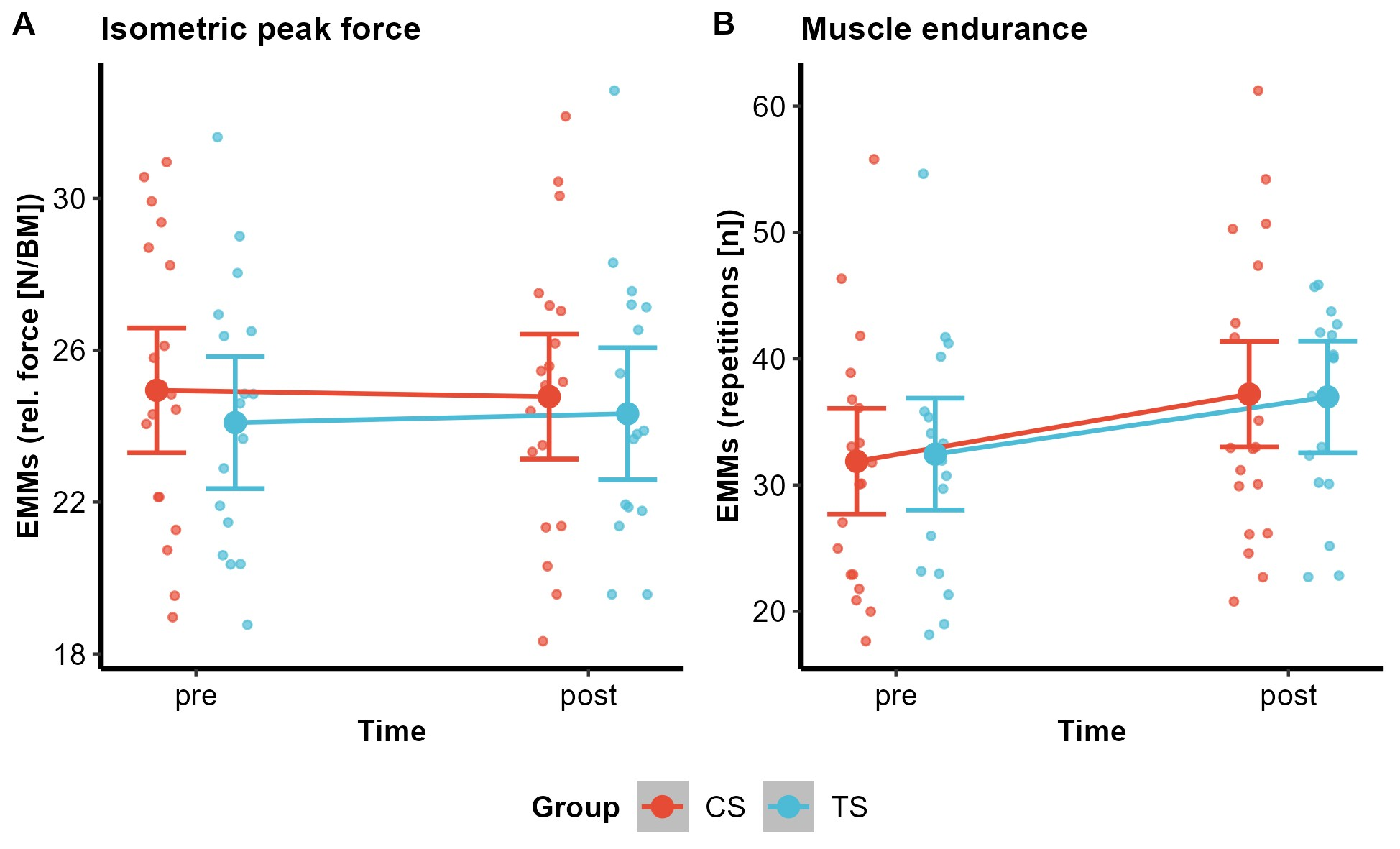


Figure S5 Estimated marginal means (EMMs) with 95% confidence intervals for relative isometric peak force (A) and maximal repetitions representing muscle endurance (B) from pre- to post-intervention for cluster set (CS) and traditional set (TS) groups.
